# Loot

**Published:** 1868-04-15

**Authors:** 


### *LOOT.*

***"Milk for Babes."***

Dr. Tracy, of Andover, Mass., writes thus of "Milk for Babes," to the Boston *Journal of Chemistry :*

"Writers generally speak as if all cows' milk was equally good, or nearly so, for babes. I wish to say that, about twelve or thirteen years ago, I was led to notice that cows' milk which retained its color after the cream had been taken off, would sit well upon the stomach; while that which was either bluish or greenish after the cream was taken off, would uniformly disagree. For the last dozen years, I have not met with a single instance where an infant has failed to be well nourished, and to flourish well, upon such milk as first mentioned; nor have I met with a single instance where one fed upon such as last named, did not suffer from colic, green dejections, and general ill health. The rule is, the less change of color between the new milk and the same milk after standing twelve hours and having the cream removed, the better the milk for infants; and, on the contrary, the greater the change of color, the more unsuitable for their purpose. This is an item that I have never seen noticed by any writer, excepting by the author of '*The Mother and her Offspring.*' No analysis is necessary to indicate to mothers the kind of milk their children require. The color after the removal of the cream, will indicate its positive or relative fitness or unfitness for children's food."

Germain to the same subject, Mr. Horsley, analyst to the county of Gloucester, in a paper on the subject, *Chemical News*, says that a milk may be of high density, and yet give but comparatively little animal matter, such as cream and caseine, whilst the amount of lactine retained in solution in the whey may be greater than usual. On the other hand, a sample of milk may be of lower density, and yet yield far more animal matter than ordinary, though each may be perfectly genuine; the difference in the relative value of the constituents depending much on the time of year, the mode of keeping and feeding the cow, etc. He found only one degree of difference between a sample purchased at Cheltenham and a sample supplied to the workhouse; but an analysis of the two specimens shows not only a vast difference in the amount of solid matter, but also that very little reliance can be placed in any of the instruments usually employed in determining the value of milk; for the fatty matter of the milk, unlike any other aqueous solution, helps to keep up the instrument, and gives no idea of the actual density of the sample, nor of its value.

In the cases of infantile intestinal trouble, where *good* milk fails to agree, much advantage will be gained by adding to it a few grains of the *Phosphate of Soda.* It is vastly better than the old-fashioned plan of giving *Hyd. cu. Cret.* every time the infantile "bowels are out of order." This is an old remedy for this sort of trouble, and a very good one; and we are happy to see Dr. Stephenson, of Edinburgh, recalling attention to it. He says:

"In infants who are being artificially reared, and who are liable to frequent derangement of the bowels; also, when the phosphatic elements in the food seem deficient, or when articles of food rich in phosphates, such as oat-meal, disagree; where, from the character of the motions, there is a deficient or defective secretion of bile. It is thus of service in cases of chalky stools, or white fluid motions. I have also found it of service in many cases of green-stools. In diarrhœa, generally, it is more difficult to distinguish the class of cases. In simple diarrhœa, such as we frequently meet with in the summer months, I have not found it of much service alone, although it may be of use when given in combination with other remedies. It is chiefly in that class of cases which are more properly termed duodenal dyspepsia, that it is of benefit. Diarrhœa after weaning is generally of this nature, and the cases are often chronic, or of some weeks' standing; the mother generally having exhausted her own and the nearest druggist's resources, before applying for advice. It is also of service in some cases where the diarrhœa is due to some general cachexia."

He also uses it with adults in some cases of constipation, and in cases of duodenal dyspepsia. He likens its action in phthisis to that of the hypophosphites of soda. The dose for children is four to ten grains, in the food; for adults, twenty to forty grains, in water, and taken after meals. Too small doses fail of their action.

### *Decolorizing Tinct. Iodine.*

Some time since, we gave in the JOURNAL a formula for decolorizing *Tinct. Iodine*, by the addition of a few drops of *Carbolic acid*—giving a solution of "*Carbolate of Iodine.*" Dr. Baruch, of S. C., writes to the *Medical Record* that sometimes this may be contra-indicated. In such cases, he observes:

"I have ascertained that the *Hyposulphite of soda* has the peculiar effect of depriving the *Iodine* of its color, forming a perfectly limpid fluid, which does not form the purple *Iodide of starch* on the linen, nor produce a yellow discoloration of the skin. The *Hyposulphite* having no medicinal effect in the small quantity required for this purpose, the *Tr. iodine* suffers no addition to nor detraction from its therapeutic properties. I make a saturated solution of the *Hyposulphite* in water, and this is added in proportion of about one-sixth of the *Tr. Iodine*. The latter floats upon the former; but, through agitation, will produce a beautifully clear solution with the properties mentioned. The solution of the *Bisulphite* answers the same purpose. If we desire to obtain the effect of the undiluted *Tr. iodine*, we need only dissolve a few small crystals of the *Hyposulphite*, or a little of the powder of the *Bisulphite*, in the tincture, and complete decoloration will be the result.

Among the varied uses to which *Carbolic acid* is being put, Dr. Waring commends its addition to zinc ointment, in badly smelling sores, cancer, etc. Five grains to the ounce of zinc ointment, is recommended as a local application to fœtid feet and armpits.

### *Treatment of Venereal Diseases in Paris.*

Dr. Yandell, writing from Paris to the *Nashville Journal*, says that in the *Hospital du Midi* they insist on puncturing buboes perpendicularly, and not horizontally; the contraction of the skin thus keeping the incision open. All sorts of chancres are treated by frequent applications of *Lunar caustic*, and dressings of aromatic wine or aromatic vinegar. In Paris, as in London, "a bewildering contrariety of opinion" exists as to the varieties or identities of syphilis; and many even contend that syphilis sometimes results from a blenorrhœa. Circumcision is resorted to, whenever any excuse is afforded — prominently as "security for the future," after a very French fashion.

*Cubebs* and *Copaiba* maintain their pristine ascendancy in the treatment of blenorrhagia, to such an extent that many patients prefer to submit to the disease rather than suffer the nauseous remedy. It is scarcely probable that in the treatment of venereal diseases, "they do these things better in France" than in this country. In fact, no man can transplant the details of French therapeutics to this country, and succeed in practice. The best part of their therapeutics is their cookery, which Dr. Y. strongly commends; incidentally observing, "Bad cooking, I am convinced, killed more Confederate soldiers than were killed by the Yankees." The millenium can not come, of course, until sin ceases in the world; but sin can never disappear, while the brains and nerves of men are tortured by unwholesome blood, the result of bad cooking; and so I conclude that the man who improves the cookery of his country, is one of its true benefactors — certainly the peer of him who makes 'two blades of grass to grow where one grew before.'" All of which, we steadfastly believe.

### *Preserving Anatomical Specimens.*

The *Journal de Pharmacie et de Chirurgie* records the process of M. Van Vetter, of the Anatomical Museum of the University of Boulogne, in preserving anatomical specimens:

He takes seven parts of glycerine, one part of brown sugar, and half a part of *Nitre*, and mixes them. The pieces to be preserved are placed in this, and allowed to remain until hard as wood. The time depends upon their size — a hand requiring eight days. They are then suspended in a dry, warm apartment. The glycerine soon evaporates, leaving the specimens soft and supple, and with their natural color. The action and mechanism of the muscles may be demonstrated from specimens thus prepared.

### *Cannabis Indica*

Is again spoken of in the treatment of delirium tremens.

---

### AMERICAN MEDICAL ASSOCIATION.

*Office of Permanent Secretary*, Wm. B. Atkinson, M.D., *S. W. Cor. Broad and Pine Sts., Philadelphia.*

The Nineteenth Annual Meeting of the American Medical Association will be held in Washington, on Tuesday, May 5th, 1868, at 11 o'clock A.M.

The following committees are expected to report:

On Ophthalmology, Dr. J. S. Hildreth, Illinois, Chairman.

On Cultivation of the Cinchona Tree, Dr. J. M. Toner, D. C., Chairman.

On Surgical Diseases of Women, Dr. Theophilus Parvin, Ind., Chairman.

On Rank of Medical Men in the Navy, Dr. N. S. Davis, Ill., Chairman.

On Insanity, Dr. C. A. Lee, N. Y., Chairman.

On American Medical Necrology, Dr. C. C. Cox, Md., Chairman.

On Leakage of Gas Pipes, Dr. J. C. Draper, N. Y., Chairman.

On Medical Ethics, ——— ———, Chairman.

On Plan of Organization, Dr. C. C. Cox, Md., Chairman.

On Provision for the Insane, Dr. C. A. Lee, N. Y., Chairman.

On the Climatology and Epidemics of Maine, Dr. J. C. Weston; of New Hampshire, Dr. P. A. Stackpole; Vermont, Dr. Henry Janes; Massachusetts, Dr. Alfred C. Garrett; Rhode Island, Dr. C. W. Parsons; Connecticut, Dr. E. K. Hunt; New York, Dr. W. F. Thoms; New Jersey, Dr. Ezra M. Hunt; Pennsylvania, Dr. D. F. Condie; Maryland, Dr. O. S. Mahon; Georgia, Dr. Juriah Harriss; Missouri, Dr. Geo. Engelman; Alabama, Dr. R. Miller; Texas, Dr. T. J. Heard; Illinois, Dr. R. C. Hamill; Indiana, Dr. J. F. Hibbard; District of Columbia, Dr. T. Antisell; Iowa, Dr. J. W. H. Baker; Michigan, Dr. Abm. Sager; Ohio, Dr. J. W. Russell; California, Dr. F. W. Hatch; Tennessee, Dr. Joseph Jones; West Virginia; Dr. E. A. Hildreth; Minnesota, Dr. Samuel Willey.

On Clinical Thermometry in Diphtheria, Dr. Jos. G. Richardson, N. Y., Chairman.

On the Treatment of Disease by Atomized Substances, Dr. A. G. Field, Iowa, Chairman.

On the Ligation of Arteries, Dr. Benj. Howard, N. Y., Chairman.

On the Treatment of Club-Foot without Tenotomy, Dr. L. A. Sayre, N. Y., Chairman.

On the Radical Cure of Hernia, Dr. G. C. Blackman, Ohio, Chairman.

On Operations for Hare-Lip, Dr. Hammer, Mo., Chairman.

On Errors and Diagnosis in Abdominal Tumors, Dr. G. C. E. Weber, Ohio, Chairman.

On Prize Essays, Dr. Chas. Woodward, Ohio, Chairman.

On Medical Education, Dr. A. B. Palmer, Mich., Chairman.

On Medical Literature, Dr. G. Mendenhall, Ohio, Chairman.

Secretaries of all medical organizations are requested to forward lists of their Delegates as soon as elected, to the Permanent Secretary. W. B. Atkinson.

---

### *Receipts since Jan. 1.*

The following is a list of persons from whom money has been received at this office for the Journal, together with the amount paid by each, since the beginning of the current year :

Annis, T E, $2; Armstrong, J M, 6; Albin, G W, 3; Armstrong, J S, 2; Arter, J R, 2; Allen, C C. 3; Amos, E, 3; Armstrong, L G, 3; Armstrong, W G, 10; Adams, H D, 3; Andrews, L M, 4.50; Allen, C I, 3; Adams, O B, 3; Anthony, J P, 3.

Barr, J A, 2; Broughton, R, 2.25; Barnwell, J H, 5; Bosco, O H, 2; Bradbury, W T, 5; Boole. T N, 3; Blood, S, 2; Berchelman, A, 3; Barkett, B, 2; Burner, S A, 6; Bingham, R H, 3; Bates, J H, 2; Bacon, A J, 3; Boening, L, 3; Booth, 3; Brigham, J W, 3; Brookhart, L, 5; Baker, J H, 2; Bailey, W W, 2; Barnett, J R, 2; Baldwin, M O, 4.50; Brown, L, 2; Bennett, J, 3; Boardman, J G, 3.50; Blanchard, J G, 3; Brown, J Z; 4.50; Boardman, E R, 3; Bradshaw, B H, 6; Barnes, R A, 3; Baker, B, 3; Bayles, E W, 3; Bailey, F K, 3; Brown, J, 5.

Corkins, P G, 6; Cozad, J, 3; Coover, W H, 3; Covert, G W, 2; Cory, J B, 3; Cusack, J H, 5; Clark, S, 3; Cowden, J W, 2; Chase, H A, 2; Crosswait, P R, 3; Cook, J A, 2; Crane, W H, .75; Clark, W W, 3; Cole, F, 3; Campbell, J W, 2; Clark, J A, 3; Chesbrough, H F, 3; Comstock, T G, 3; Cargen, W, 3; Cook, E P, 3; Clark, H N, 3; Carlisle, C H, 2.20; Cloud, J B, 3; Cole & Crosswait, 3; Condon & McClelland, .75; Clark, Ossian, 6.

Drake, J T, 4; Dunn, J W, 3; Dietrich, T A, 3; Durr, J A, 10; Davidson, D L, 2; Davis, B F, 3; Dora, J W, 3; Dunn, A A, 3; Drummond & Bower, 1; Douglas, A C, 3; Drake, N A, 2; Dyer, L, 3; Danforth, I N, 3; Dant, J R, 5.

Elhardt, F, 2; Elder, T A, 2; Egbert, J B, 6; Ellis, D E, 3.

Fawcett, H B, 4; Felker, J B, 6; Flynn, W, 2; Fisk, G, 2; Fenton, S C, 3; Foster, J H, 8; Fenn, C T, 3; Fish, S N, 3; Fifield, F, 2: Flood. J R, 3; Ford, C L, 3.

Gibbs, J A M, 2; Gill, C J, 3; Gibson, A C, 2: Galloway, S, 8; Gray, J D, 6; Grattan, E H, 3; Griffith, J H, 3; Goldsbury, J A, 3; Gandy, J L, 3; Goodell, J H, 1.50; Glassner, E T, 6; Garven, 5; Gray, S C, 7; Galer, J B, 5; Green, J S, 8.

Heavenridge, A, 3: Hughes, M, 2; Horton, H E, 3; Hall, R W, 3; Hoffman, J E, 2; Hollingsworth, 5; Hain, W F, 2; Haskell, A S, 3; Hagey, W H, 2; Herdman, J W, 3; Holland, A, 1.25; Hutchins, J H, 2; Hodge, A, 1; Hutchings, 4; Hagellsen, 3; Hobbins, J H, 6; Hulburt, V L, 3; Haggard, J R, 2; Hart, H W, 3; Hayes, J, 3; Hait, C E, 8; Hopkins, R, 8; Herman, P J, 3; Hunt, T, 6; Hoss, J V, 3; Hunt, J, 3; Henry, A F, 3; Haladay, A, 3.

Irwin, E H, 3; Ireland, A B, 3.

James, O, 2; Johnston, Thomas, 3; Jones, J R, 3; Johnson, C J, 5.50.

Kelsey, W J, 3; Kirkpatrick, G W, 5; Kimball, 3; Keiper, G F, 3; Kinnear, A H, 3; Knowles, J, 3; Kennicott, 3; Knox, W A, 3; Keigan, C J, 2; Kennedy & Moor, 10; Kyner, D J, 6.

Lyman, M J, 3; Love, H, 6; Lueck, A W, 3; Latta, M M, 6; Lewis, J B W, 3; Little, J, 1; Leffingwell, J W, 3; Leavitt, J J, 2; Layne, P M, 4; Long, S O, 3; Leonard, W R, 2; Low, J, 3; Law, W, 6.

Mowrer, P A, 4.50; Morrow, G M, 2; May, W L, 5; McCleary, 5; Mead, A J, 3; Maloney, J M, 2; Mahon, 7: Miller, J W, 6, Mansen, W, 2; Middleditch, A, 3; Messner, L C, 2; Murphy, S C, 3; Montgomery, D B, 2; Marshall, E B, 2; McKune, A B, 2; Marshall, W R, 3; Miller, A, 2; Moffitt, J, 2; McCulloch, A P, 2; McCaughey, T C, 2; Mayo, E L, 2; Moore, R L, 3; McClure, A W, 3; Meek, J A, 6; Miller, A J, 3; Morse, W A, 3; Monroe, H, 3; Mergler, 3; McFarland, P M, 1; McKnight, W, 3; Molony, R S, 6; Martin, F E, 2; Moore, A V, 2; Mallett, 6.50; May, J K, 6; Montgomery, D B, 1; Murphy, Thomas, 2.

Newkirk, G, 2; Nelson, A P, 6.

O'Brien, J H, 5.

Patton, W R, 2; Parks, T H, 3; Pankhurst, 2; Pierce, R W, 5; Peters, J H, 2; Plummer, S C, 6; Pratt, A S, 6; Patton, W K, 3; Porter, W B, 3; Price, W H, 3; Perry, J W, 3; Prentice, J, 3.

Ray, R B, 6; Ross, B F, 6; Rea, D, 8; Riddler, J G, 5; Reed, C B, 2.50; Rankin, J M, 1.50; Redmon, L, 3; Rush, B H, 3; Reed, R, 2; Robinson, J. H. 10; Riddle, H R, 2; Rusell, E F, 3; Robinson, S E, 3; Rodman, A J, 6; Rich, K E, 3.

Sweetland, W M, 3; Salisbury, S S, 3.50; Sigworth, H W, 3; Somers, W, 3; Smith, A E, 3; Sims, J, 5; Sprague, C C, 3; Smith, C H, 3; Stevens, D R, 3: Smith, W H H, 2; Simpson, J, 2; Searles, J, 1; Skeer, J D, 3; Snyder, J D, 3; Spitler, E, 2; Seely, O F, 2; Saucerman, J M, 3; Silverthorn, L L, 5; Sherman, N, 3; Sullivan, W H, 5; Smith. H W. 1; Smith, M F, 3.

Trueworthy, J W, 2; Tuttle, J V, 3; Thayer, J W, 3; True, B F, 3; Trabue, J W, 3; Tebo, G H, 3; Taylor, G O, 2; Thompson, D D, 2; Tucker, B B, 3; Thurman, J B, 3; Towne & Brett, 8; Tucker, J B, 3.

Vosburg, D M, .75; Van Nus, J S M C, 3; Van Meter, H, 3.

Williams, J D, 2; Whittlesey, S H, 2; Waterhouse, M, 5; Webstee, J R, 3; Worsley, G, 3; Walsten, R L, 3; Wheaton, 2; Winson, J, 3.25; Warner, H, 3; Witherspoon, M V B, 1.25; Williams, J A, 3; Webster, J C, 2; Woodworth, L C, 3; Wetmore, R B, 3; Wood, T F, 1.50; Webster, B M, 2; Wheeler, E N, 2; Wilson, W T, 3; Woodman, E, 2.

Yount, T J, 2.

